# Extraction and Oxford Nanopore sequencing of genomic DNA from filamentous Actinobacteria

**DOI:** 10.1016/j.xpro.2022.101955

**Published:** 2022-12-16

**Authors:** Maria Alvarez-Arevalo, Eva Baggesgaard Sterndorff, David Faurdal, Tue Sparholt Jørgensen, Anna-Sophie Mourched, Oliwia Vuksanovic, Subhasish Saha, Tilmann Weber

**Affiliations:** 1 The Novo Nordisk Foundation Center for Biosustainability, Technical University of Denmark (DTU), Kemitorvet, 2800 Kgs. Lyngby, Denmark

**Keywords:** Genomics, Sequencing, Microbiology, Molecular Biology

## Abstract

Actinomycetota (Actinobacteria) is an ecologically and industrially important phylum which is challenging to extract pure high-molecular-weight (HMW) DNA from. This protocol provides a parallelized, cost-effective, and straightforward approach for consistently extracting pure HMW DNA using modified non-toxic commercial kits suitable for higher throughput applications. We further provide a workflow for sequencing and assembly of complete genomes using an optimized Oxford Nanopore rapid barcoding protocol and Illumina data error correction.

## Highlights

• Extraction and purification of HMW DNA in filamentous Actinobacteria
• A cost-effective parallelized approach for handling heterogeneous samples
• Dual parallel sequencing using Nanopore and Illumina platforms
• Assembly of high-quality whole genomes *de novo*

## Abstract (editor highlights)

Publisher’s note: Undertaking any experimental protocol requires adherence to local institutional guidelines for laboratory safety and ethics.

## Graphical abstract

**Figure:**
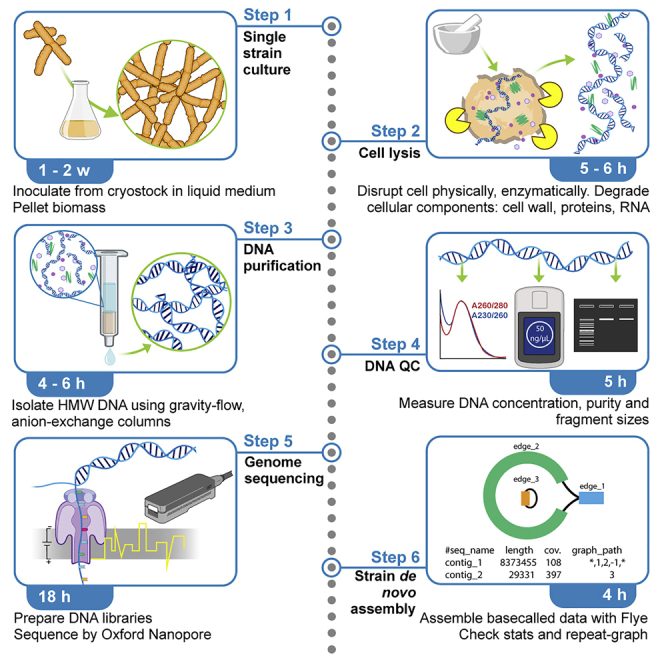

## Teaser

Actinomycetota (Actinobacteria) is an ecologically and industrially important phylum which are challenging to extract pure high-molecular-weight (HMW) DNA from. This protocol provides a parallelized, cost-effective, and straightforward approach for consistently extracting pure HMW DNA using modified non-toxic commercial kits suitable for higher throughput applications. We further provide a workflow for sequencing and assembly of complete genomes using an optimized Oxford Nanopore rapid barcoding protocol and Illumina data error correction.

## Before you begin

This protocol describes the necessary steps for the extraction, purification and sequencing of HMW DNA from soil-derived filamentous Actinobacteria. The protocol has been successfully used on the following actinobacterial genera, based on GTDB-tk^1^: *Actinoallomurus, Actinomycetospora, Actinophytocola, Actinoplanes, Actinosynnema, Aldersonia, Amycolatopsis, Curtobacterium, Dactylosporangium, Embleya, Kineococcus, Kitasatospora, Kocuria, Kribbella, Lentzea, Microbispora, Micrococcus, Micromonospora, Microbispora, Microtetraspora, Mycobacterium, Nocardia, Nocardioides, Nocardiopsis, Nonomuraea, Pseudonocardia, Rhodococcus_C, Saccharomonospora, Saccharopolyspora, Sphaerisporangium, Spirillospora, Streptomyces, Streptomyces_B, Streptosporangium, and Williamsia_A*. The protocol has also inadvertently proved successful for the following non-actinobacterial genera: *Bacillus, Enterococcus_B, Niallia, Staphylococcus, Bosea, Methylobacterium*, and *Pseudomonas*.

The first part of the protocol is optimized from the QIAGEN genomic-tip extraction protocol^2^, where increased incubation times and mechanical treatment efficiently lyses the cells. The second part of the protocol describes the steps for sequencing by Oxford Nanopore using the SQK-RBK110.96 kit for rapid barcoding, optimized for running 12–16 genomes on a MinION flow cell in ∼ 24 h with an increased read length compared to the original protocol. The third part of the protocol describes assembly, polishing, and quality control (QC) of the finished genomes.

Before beginning, buffer, media, and enzyme solutions must be prepared or purchased. Before Nanopore sequencing, MinKNOW must be installed to run the MinION flow cells. For assembly and visualization, Flye^3^ and Bandage^4^ should be installed. Before polishing and QC of the complete genome to produce a high-quality genome, Polypolish,^5^ POLCA^6^ and BUSCO^7^ needs to be installed.

All software was run on a Debian machine with a GFORCE RTX 2080 GPU with 8 GB RAM. Guidelines and computer requirements for running MinKNOW are provided by Oxford Nanopore (https://nanoporetech.com/community/lab-it-requirements) and relevant benchmarks are provided for Flye in its GitHub repository (https://github.com/fenderglass/Flye/blob/flye/docs/USAGE.md#performance).

### Preparation: Software

The software indicated in Table 1 must be installed to perform the sequencing, basecalling, assembly and polishing described in this protocol.

**Table 1:** List of software, installation links and their purposes

Software	Installation link	Purpose
MinKNOW	https://community.nanoporetech.com	Sequencing, Guppy for GPU basecalling, Guppy for GPU demultiplexing
Flye	https://github.com/fenderglass/Flye	Genome assembly
Polypolish	https://github.com/rrwick/Polypolish	Genome assembly polishing
POLCA (MaSuRCA)	https://github.com/alekseyzimin/masurca	Genome assembly polishing
Bandage	https://github.com/rrwick/Bandage	Genome assembly repeat graph viewer
Filtlong	https://github.com/rrwick/Filtlong	Nanopore reads filtration
BUSCO	https://gitlab.com/ezlab/busco/-/releases#5.4.3	Genome assembly quality evaluation

## Key resources table

**Table:** 

REAGENT or RESOURCE	SOURCE	IDENTIFIER
**Chemicals, peptides, and recombinant proteins**		

MilliQ water	N/A	N/A
Lysozyme from chicken egg white, lyophilized powder	Sigma-Aldrich	Cat #L6876
RNAse A	QIAGEN	Cat #19101
Proteinase K	Invitrogen	Cat #25530049
Disodium ethylenediaminetetraacetate dihydrate	Sigma-Aldrich	Cat #E5134
Tris-HCl, pH 7.4 1 M	Thermo Fisher Scientific	Cat #J22638.AP
Trizma base	Sigma-Aldrich	Cat #93362
Tween 20	Sigma-Aldrich	Cat #P9416
Triton X-100	Sigma-Aldrich	Cat #93443
Buffer QBT	QIAGEN	Cat #19054
Buffer QC	QIAGEN	Cat #19053
Buffer QF	QIAGEN	Cat #19055
Ethanol, absolute	VWR Chemicals	Cat #20821.310
Isopropanol	VWR Chemicals	Cat #20880.290
Ethanol 70%	VWR Chemicals	Cat #83801.360
Tap water	N/A	N/A
Oxoid™ Malt Extract	Thermo Fisher Scientific	Cat #LP0039B
Difco™ Yeast Extract	Thermo Fisher Scientific	Cat #210934
N-Z-Amine® A	Sigma-Aldrich	Cat #C0626
Bacto™ Peptone	Thermo Fisher Scientific	Cat #211677
Meat extract	Sigma-Aldrich	Cat #70164
Soluble starch from potato	Sigma-Aldrich	Cat #S2004
D-(+)-Glucose monohydrate	Thermo Fisher Scientific	Cat # A11090.36
Dextrose	Sigma-Aldrich	Cat #D9434-1KG
BD Difco™ Dehydrated Culture Media: ISP Medium 2	Fischer Scientific	Cat #BD 277010
Phosphate-buffered saline, pH 7.2, liquid, sterile-filtered	Sigma-Aldrich	Cat #806544
BD Difco™ Dehydrated Culture Media: ISP Medium 2	Fisher Scientific	Cat #BD 277010
Magnesium chloride hexahydrate	Sigma-Aldrich	Cat #M2670
Glycine	Sigma-Aldrich	Cat #G8790
Agar	Sigma-Aldrich	Cat #A1296
D-Mannitol	Sigma-Aldrich	Cat #M4125
Fat reduced soya flour	Hensel	Cat #02004307
Guanidine hydrochloride	Sigma-Aldrich	Cat #G3272
Dry ice	N/A	N/A
Liquid nitrogen	N/A	N/A
UltraPure™ DNase/RNase-Free Distilled Water	Thermo Fisher Scientific	Cat #10977035
TAE Buffer (Tris-acetate-EDTA) (50×)	Thermo Fisher Scientific	Cat #B49
RedSafe™ Nucleic Acid Staining Solution	iNtRON Biotechnology	Cat #21141
TopVision Agarose	Thermo Scientific™	Cat #R0492
Quick-Load 1 kb Extend DNA Ladder	New England Biolabs	Cat #NEB-N3239S
Gel loading dye 6×	NEB	Cat #B7021S
Hydrochloric acid	Sigma-Aldrich	Cat #30721-1L-M
Sodium hydroxide	Sigma-Aldrich	Cat #S8263-150ML

**Critical commercial assays**		

QIAGEN Genomic Tip 20/G	QIAGEN	Cat #10223
Qubit dsDNA Broad Range Assay Kit	Invitrogen	Cat #Q32853
Rapid Barcoding Kit	Oxford Nanopore	Cat #SQK-RBK110.96
Short Read Eliminator Kit	Circulomics	Cat #SKU SS-100-101-01
KAPA Pure Beads	Roche	Cat #07983271001
Leica Microsystems Immersion Oil for Microscopes type F	Leica Microsystems	Cat #11513859

**Experimental models: Organisms/strains**		

Actinobacteria strains	User	N/A

**Software and algorithms**		

Computer (requirements for MinKNOW are provided by Oxford Nanopore; for assemblies specified in Flye documentation)	N/A	https://nanoporetech.com/community/lab-it-requirements
MinKNOW, with Guppy GPU version >5	Oxford Nanopore Technologies	https://community.nanoporetech.com
Bandage	N/A	https://github.com/rrwick/Bandage
Flye	N/A	https://github.com/fenderglass/Flye
Polypolish	N/A	https://github.com/rrwick/Polypolish
POLCA	N/A	https://github.com/alekseyzimin/masurca
BUSCO	N/A	https://busco.ezlab.org/
Nanodrop 2000 / 2000c Operating Software, version 1.6	Thermo Fisher Scientific	N/A
Leica Application Suite LAS v4.12 Software	Leica	https://www.leica-microsystems.com/products/microscope-software/p/leica-application-suite/

**Other**		

Mortar and pestle	Thomas Scientific	Cat #1201U69
Spatula	Sigma-Aldrich	Cat #S4272-1EA
Liquid nitrogen container	KGW Isotherm	Cat #10222
Ice pan	VWR	Cat #216-1093
Centrifuge tube 50 mL	Sigma-Aldrich	Cat #CLS430828
Centrifuge tube 15 mL	Sigma-Aldrich	Cat #CLS430791
Erlenmeyer flasks with 4 baffles, 300 mL	Glasgerätebau Ochs	Cat #100300
1,5 mL LoBind Eppendorf Tubes	Eppendorf	Cat #0030108051
Blue cap flask 1 L	VWR	Cat #215-1595
Autoclaved toothpicks	N/A	N/A
Multipette E3	Eppendorf	Cat # 4987000010
Combitips advanced 50 mL	Eppendorf	Cat #0030089693
DynaMag™-2 Magnet – Magnetic rack for 1,5 mL tubes	Invitrogen	Cat #12321D
Biotix pipette filter tips 10 μL	VWR	Cat #613-2814
Pipette filter tips 20 μL		Cat #613-0988
Biotix pipette filter tips 200 μL	VWR	Cat #613-1378
Biotix pipette filter tips 300 μL	VWR	Cat #613-1379
Biotix pipette filter tips 1,250 μL	VWR	Cat #613-2436
Mline® Mechanical Pipette, Single Channel 0,1–3 μL	Sartorius	Cat #725010
Mline® Mechanical Pipette, Single Channel 0,5–10 μL	Sartorius	Cat #725020
Mline® Mechanical Pipette, Single Channel 2–20 μL	Sartorius	Cat #725030
Mline® Mechanical Pipette, Single Channel 10–100 μL	Sartorius	Cat #725050
Mline® Mechanical Pipette, Single Channel 20–200 μL	Sartorius	Cat #725060
Mline® Mechanical Pipette, Single Channel 100–1000 μL	Sartorius	Cat #725070
Mline® Mechanical Pipette, 12 Channel 0,5–10 μL	Sartorius	Cat #725220
Eppendorf microcentrifuge tubes 2 mL	VWR	Cat #211-2120
Thin-walled PCR tubes	Hounisen	Cat #72.991.002
MinION	Oxford Nanopore Technologies	https://store.nanoporetech.com/eu/minion.html
Flow Cell (R9.4.1)	Oxford Nanopore Technologies	Cat #FLO-MIN106D
Qubit 4 Fluorometer	Thermo Fisher Scientific	Cat #Q33238
NanoDrop™ 2000/2000c Spectrophotometers	Thermo Fisher Scientific	Cat #ND-2000
Ultrospec 10 Cell Density Meter	Amersham Biosciences	Cat #80-2116-30
Wide Mini ReadySub-Cell GT Horizontal Electrophoresis System, for precast gels	Bio-Rad	Cat #1704489
Wide Mini-Sub Cell GT Mini Handcasting Kit	Bio-Rad	Cat #1704497
ChemiDoc™ XRS+ System with Image Lab™ Software	Bio-Rad	Cat #1708265
Polyethersulfone membrane 0,22 μM	Sigma-Aldrich	Cat # GSWP04700
Kern PCB precision balances	Sigma-Aldrich	Cat #Z674753-1EA
Microcentrifuge, MiniStar / MiniStar blueline	VWR	Cat #521-2161
Thermo Scientific™ Sorvall™ Legend™ Micro 21R Microcentrifuge	Fisher Scientific	Cat #75002445
Thermo Scientific™ Multifuge™ X3 FR Centrifuge	Fisher Scientific	Cat #75004536
Microscope Leica DM4000 B	Leica	N/A
Petri dishes	Thermo Fisher Scientific	Cat #263991
Centrifuge tube holder 15 mL	Frederiksen Scientific	Cat #031114
Centrifuge tube holder 50 mL	Frederiksen Scientific	Cat #031116
Rack for microcentrifuge tubes	VWR	Cat #211-0204
Scissors	N/A	N/A
Thermomixer	Eppendorf	Cat #5382000015
Thermal Cycler C1000 Touch	Bio-Rad	Cat #1851196
Miniplate Spinner Labnet MPS 1000	Sigma-Aldrich	Cat #Z723649
Hula mixer	Invitrogen	Cat #15920D
Vortex Mixer – Wise Mix VM 10	WITEG Labortechnik	Cat #WITEG_14002
Labnet 311DS Environmental Shaking Incubator	VWR	Cat #VWRIS2031-13
LAF-Bench	N/A	N/A
Autoclave CERTOCLAV MULTICONTROL 2	Buch & Holm	Cat #9842030
Scissors	N/A	N/A
Syringe	VWR	Cat #613-2047
Freezer −20°C	Miele	FN12827S
Walk-in cold room +4°C	N/A	N/A
Thermo Scientific™ TSX Series Ultra-Low Freezer	Fisher Scientific	Cat #16647352
Nitrile-free gloves	N/A	N/A
Safety goggles	N/A	N/A
Safety gloves	N/A	N/A

## Materials and equipment

### Culture media recipes

**Table:** Solid ISP Medium 2 recipe

Reagent	Final concentration	Amount
Dehydrated culture medium ISP2	38 g/L	38 g
Tap water	N/A	250 mL
MilliQ	N/A	750 mL
**Final volume**	N/A	1,000 mL

Sterilize by autoclaving at 115°C for 15 min. Storage the media at room temperature (∼ 24°C) for up to 6 months.

**Table:** Liquid ISP Medium 2 recipe

Reagent	Final concentration	Amount
Yeast extract	4 g/L	4 g
Malt extract	10 g/L	10 g
Dextrose	4 g/L	4 g
Tap water	N/A	250 mL
MilliQ	N/A	750 mL
**Final volume**	N/A	1,000 mL

Sterilize by autoclaving at 115°C for 15 min. Storage the media at room temperature (∼ 24°C) for up to 6 months. This recipe is modified from Shirling and Gottlieb.^8^

**Table:** Liquid Yeast Extract – Malt Extract (YEME) 3.4% sucrose Medium recipe

Reagent	Final concentration	Amount
Yeast extract	3 g/L	3 g
Peptone	5 g/L	5 g
Malt extract	3 g/L	3 g
D-(+)-Glucose monohydrate	10 g/L	10 g
Sucrose	42.5 g/L	42.5 g
MilliQ	N/A	Up to 1,000 mL
MgCl_2_ ∗ 6 H_2_O 2.5 M	5 mM	2 mL
Glycine 20%	0.5% (v/v)	25 mL
**Final volume**	N/A	1,000 mL

Sterilize by autoclaving at 115°C for 15 min.

Sterile Magnesium chloride hexahydrate (MgCl_2_ ∗ 6 H_2_O) and Glycine are added after autoclaving the media. Storage the media at room temperature (∼ 24°C) for up to 6 months.

This recipe is modified from Kieser et al.^9^

**Table:** Solid Mannitol Soya (MS) Medium recipe

Reagent	Final concentration	Amount
Agar	20 g/L	20 g
Mannitol	20 g/L	20 g
Fat reduced soya flour	20 g/L	20 g
Warm tap water	N/A	Up to 1,000 mL
NaOH 5 M	0.5 mM	0.1 mL
**Final volume**	N/A	1,000 mL

Sterilize by autoclaving at 115°C for 15 min. Storage the media at room temperature (∼ 24°C) for up to 6 months. This recipe is modified from Kieser et al.^9^

**Table:** Liquid ATCC-2 pH 7.0 Medium recipe

Reagent	Final concentration	Amount
Soluble starch from potato	20 g/L	20 g
Dextrose	10 g/L	10 g
N-Z Amine® A	5 g/L	5 g
Meat extract	3 g/L	3 g
Bacto™ peptone	5 g/L	5 g
Yeast extract	5 g/L	5 g
MilliQ	N/A	Up to 1,000 mL
NaOH	N/A	variable
**Final volume**	N/A	1,000 mL

Adjust the pH to 7.0 with sodium hydroxide.

Sterilize by autoclaving at 115°C for 15 min. Storage the media at room temperature (∼ 24°C) for up to 6 months. This recipe is modified from ATCC.^10^

### Buffers and solutions

**Table:** 10 mM Tris-HCl pH 8.0 recipe

Reagent	Final concentration	Amount
Tris HCl pH 7.4 1 M	10 mM	1 mL
DNase/RNase-Free Distilled Water	N/A	99 mL
NaOH	N/A	variable
**Final volume**	N/A	100 mL

Adjust the pH to 8.0 with sodium hydroxide. Storage the solution at 4°C for up to 6 months.

**Table:** Lysozyme 100 mg/mL recipe

Reagent	Final concentration	Amount
Lysozyme from chicken egg white, lyophilized powder	100 g/L	2.5 g
MilliQ water	N/A	25 mL
**Final volume**	N/A	25 mL

Sterilize by filtration (0.20 μm syringe filter). Storage the solution at −20°C for up to 6 months.

**Table:** Buffer B1 recipe

Reagent	Final concentration	Amount
Na_2_EDTA ∗ 2H_2_O	50 mM	18.61 g
Trizma base	50 mM	6.06 g
Tween-20 10% solution	0.5% (v/v)	50 mL
Triton X-100 10% solution	0.5% (v/v)	50 mL
MilliQ water	N/A	Up to 1,000 mL
NaOH	N/A	Variable
**Final volume**	N/A	1,000 mL

Dissolve completely Disodium EDTA dihydrate and Trizma base in a ½ of the MilliQ water volume prior to adding Tween-20 solution and Triton X-100. Both Tween-20 solution and Triton X-100 solution are added afterwards, as they are strongly foaming agents.

Adjust the pH to 8.0 with sodium hydroxide.

Sterile by filtration using polyethersulfone membrane (0.22 μm).

Storage the buffer at room temperature (∼ 24°C) for up to 6 months.

This recipe is from QIAGEN.^2^

**Table:** Buffer B2 recipe

Reagent	Final concentration	Amount
Guanidine HCl	3 M	286.59 g
Tween-20 100%	20% (v/v)	200 mL
MilliQ water	N/A	Up to 1,000 mL
**Final volume**	N/A	1,000 mL

Dissolve completely guanidine HCl in ½ of the MilliQ water volume prior to adding Tween-20.

Sterile by filtration using polyethersulfone membrane (0.22 μm).

Storage the buffer at room temperature (∼ 24°C) for up to 6 months.

This recipe is from QIAGEN.^2^

**Table:** Buffer B3 recipe

Reagent	Final concentration	Amount
Buffer B1	74.5% (v/v)	745 mL
Buffer B2	25.5% (v/v)	255 mL
**Final volume**	N/A	1,000 mL

Storage the buffer at room temperature (∼ 24°C) for up to 6 months.

**Table:** Lysis mix recipe

Reagent	Final concentration	Amount
Buffer B1	94.93% (v/v)	3.5 mL per sample
RNAse A (100 mg/mL)	0.19% (v/v)	7 μL per sample
Lysozyme (100 mg/mL)	2.17% (v/v)	80 μL per sample
Proteinase K (20 mg/mL)	2.71% (v/v)	100 μL per sample

Storage the solution at room temperature (∼ 24°C) until use.

## Step-by-step method details

### Culturing actinobacteria

**Timing: 2–3 h (28–56 cultures) + growth (2–7 days)**

In this step, the actinobacterial culture is grown in a liquid medium to generate sufficient biomass for DNA extraction. The culture should produce a minimum of 250 mg cell pellet.

1. In a laminar air flow (LAF) bench, inoculate 50 mL of liquid ISP2 in a 300 mL baffled flask with the strain of interest from either an agar plate or directly from a cryostock.

***Note:*** This protocol has been used successfully with liquid ISP2, YEME, MS and ATCC-2.

2. Incubate the culture at 30°C and 140 rpm until the culture can yield two pellets of 250 mg each (typically 2–7 days but it may take longer).

***Note:*** The growth rate will be highly dependent on the strain and conditions. If after 14 days no growth is observed consult troubleshooting, problem 1.

### Discard contaminated cultures

**Timing: 30 min + growth**

**Figure 1:**
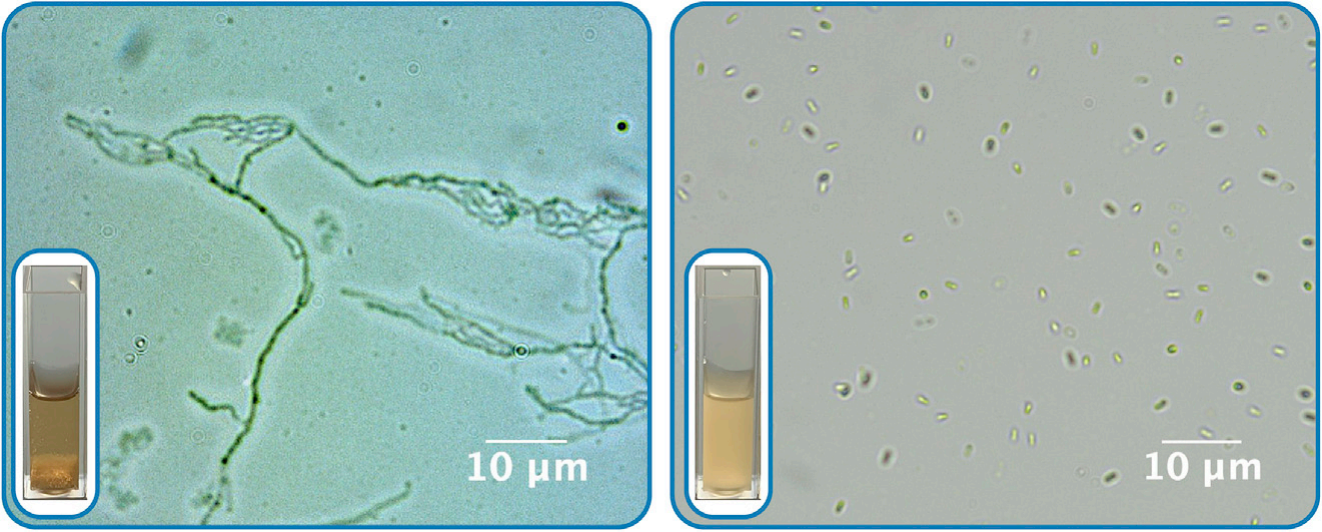
Comparison and examination of contamination under a bright field optical microscope in two different grown cultures In the left slide (1000 ×, scale bar 10 µm), a mycelial bacterial morphology characteristic of Streptomyces can be observed. In the right slide (1000 ×, scale bar 10 µm), the bacillary morphology indicates that the culture does not correspond to Actinobacteria. Each slide shows the appearance of the original culture in the lower left corner.
4. High optical density, measured as OD_600_ > 2 on a cell density meter, indicates the rapid growth of the culture within hours of inoculation.
5. Place the culture on the table and observe the sedimentation of the cells, Actinobacteria will sediment faster than *E. coli.*
6. Once sedimented, tilt the flask to one side and observe the migration of the particles. Actinobacteria move faster, whereas contaminants leave a trail behind them.
7. In a LAF bench, streak the culture on an ISP2 agar plate (with the same medium as the previous culture).
  a. After 1 or 2 days in an incubator at 30°C, observe the morphology of the colonies:

***Note:*** If the colonies have a glossy appearance, the culture may be contaminated.

Contamination often appears in the early growth phase since contaminants (such as *Escherichia coli* and *Bacillus spp*.) typically grow at a faster rate than Actinobacteria. If contamination is present, one or more of the following signs may be observed in the culture after one day of inoculation:

3. Under an optical microscope, the typical filamentous morphology of Actinobacteria is not observed (see Figure 1).

***Alternatives:*** Use a toothpick to scrape the surface, if the colony texture is slimy and slippery it is likely contaminated since most of the Actinobacteria will start to crumble or be quite difficult to pull off the surface.

### Cell harvesting

**Timing: 2–4 h (28–56 samples)**

In this step, biomass is collected from liquid culture to prepare it for DNA extraction. In the end, this should result in 250 mg of harvested cell pellet per sample.

**Figure 2:**
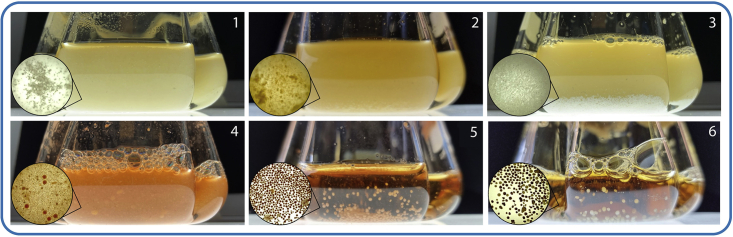
Morphological diversity of growth in Actinobacteria in liquid medium Cultures 1 and 2 correspond to a “turbid” type of growth, 3 is an intermediate culture form and would be treated as “turbid”. Cultures 4, 5 and 6 are examples of the “aggregated” type. Close-up pictures of cultures in 6-well plates are shown in circles (no magnification).
9. Transfer a 2 mL aliquot from the liquid culture into an Eppendorf tube.

***Note:*** If the culture is aggregated, it is often easier to collect the mycelium using a P1000 pipette tip with the end cut off. This will allow the biomass to enter the pipette tip.

8. Inspect the growth of your liquid culture and note whether the cells tend to grow in an aggregated or turbid manner, Figure 2.
  a. In an **aggregated culture** the cells form small lumps and are not suspended in the medium. Thus, the culture exhibits little to no turbidity instead growing in a flocculent or sedimented manner. This is a common growth pattern for Actinobacteria.
    ***Note:*** Aggregation of the cells decreases the area available to enzymes during cell wall degradation, and as such, they must be ground to powder in liquid nitrogen (LN) as part of the extraction process.
  b. In a **turbid culture** the cells grow suspended in the medium producing the opaque/cloudy cultures common for many lab-grown bacterial cultures. Since the cells are not heavily aggregated there is plenty of surface area available for enzymatic activity.

10. Centrifuge the sample at 13,000 × *g* for 1 min and discard the supernatant.
11. Add 500 μL of phosphate-buffered saline (PBS, pH = 7.4) and resuspend the pellet by vortexing for 10–20 s.
12. Centrifuge the sample again at 13,000 × *g* for 1 min and discard the supernatant. If there is little to no pellet (less than 150 mg), see troubleshooting, problem 1.
13. Store the harvested cells at −80°C at least 12 h or up to several months.

### Cell lysis

**Timing: 1–2 h hands-on + 4 h of incubation (28–56 samples)**

In this step, DNA is extracted by enzymatic lysis, while RNA and proteins are degraded by treatment with RNAse A and proteinase K. Samples that grew in an aggregated manner need to be ground to powder in LN before enzymatic treatment. As such, they take longer to process and grouping them together is advisable.

14. Prepare fresh lysis mix for the number of samples according to the lysis mix recipe in Buffers and solutions.
15. For each sample, dispense 3.5 mL lysis mix into a 50 mL Falcon tube.
16. Add the frozen cell pellet to the lysis mix, according to the culture’s mode of growth:
  a. If the sample grew aggregated, grind it into a powder in LN (see Methods video S1: Physical disruption of cellular aggregates by grinding in liquid nitrogen cooled mortar).
    i. For each sample, submerge a clean mortar and pestle in LN in a LN compatible ice pan.
      **CRITICAL:** Handle LN with appropriate safety gear (cryo-gloves, safety goggles, lab coat, closed-toe shoes, and long pants).
    ii. Once cool, remove the mortar and pestle from the ice pan.
      ***Note:*** Leaving a small amount of LN in the mortar will ensure the sample stays sufficiently cold.
    iii. Place the still frozen pellet into the mortar and grind it into a fine powder.
      **CRITICAL:** The pellet must remain at the temperature of the LN to minimize DNA shearing.
    iv. Scrape the powdered cell pellet into the Falcon tube with lysis mix.
      Methods video S1. Physical disruption of cellular aggregates by grinding in liquid nitrogen cooled mortar, related to step 16
  b. If the sample was turbid simply add the frozen bacterial pellet to the Falcon tube with lysis mix.
17. Homogenize the sample by vortexing for 10 s.
18. Incubate the sample at 37°C and 50 rpm for 2 h in a horizontal position.

***Note:*** This extended incubation allows for complete lysis of the cell walls. The speed of the shaking should not exceed 50 rpm but can be lowered to minimize DNA shearing further.

19. Add 1.2 mL Buffer B2 to each sample.
20. Incubate the sample at 50°C and 50 rpm for 2 h.

***Note:*** This extended incubation allows proteinase K to degrade cellular proteins completely.

**Pause point:** The now lysed samples can be stored at 4°C for ∼ 12 h.

### DNA purification and precipitation

**Timing: 4–6 h (28–56 samples)**

In this step, the extracted DNA is purified using QIAGEN Genomic-tip 20/G columns and afterwards precipitated via isopropanol to concentrate the DNA.

21. Place QF Buffer at 50°C until needed.
22. Vortex each sample for 10 s.
23. Centrifuge the samples at 8,000 × *g* for 10 min.

***Note:*** This pellets left-over cellular debris, which increases the purity of the extracted DNA and avoids clogging columns.

24. For each sample, place a QIAGEN Genomic-tip 20/G column into a 15 mL Falcon tube using the plastic spacer which comes with the kit.
25. Calibrate the 20/G columns with 1 mL of QBT Buffer.
26. Load 2–3 mL of the supernatant by pouring from the samples onto the columns. Take care to avoid the cell debris as it can clog the columns.

***Note:*** If possible, avoid using a pipette to transfer the liquid to minimize shearing.

27. Wait for the samples to pass through the columns (up to 20 min). If the samples do not pass through, see troubleshooting, problem 2.
28. Add 2 mL QC Buffer to each column and let it pass through.
29. Add an additional 2 mL QC Buffer to each column and let it pass through.
30. Discard the flow-through and place the columns into new clean 15 mL Falcon tubes.
31. Elute the DNA with 2 mL preheated QF Buffer.
32. Discard the columns and save the Falcon tubes with eluted DNA.

**Pause point:** The eluted samples can be stored at 4°C for ∼ 12 h.

**Figure 3:**
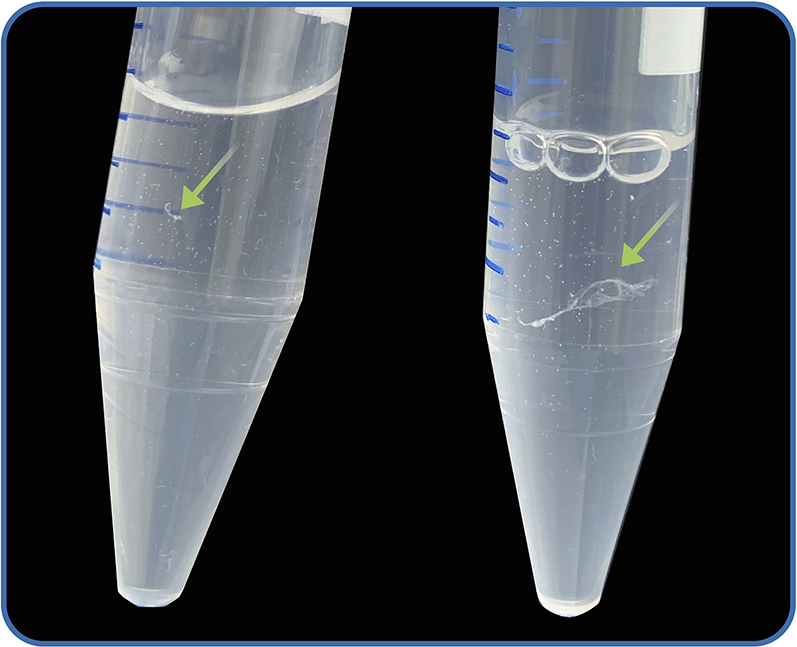
DNA following precipitation with isopropanol Green arrows point to the precipitated DNA.
36. Centrifuge the samples for 15 min at 8,000 × *g* 4°C.
37. Carefully discard the supernatant by decanting or pipetting. Then add 2 mL of ice-cold 70% ethanol.

***Note:*** Take care to avoid dislodging the pelleted DNA. The pellet might not be visible at this stage.

33. Prepare 2 mL of 70% (v/v) ethanol per sample by diluting absolute ethanol with nuclease-free water. Place the prepared ethanol on ice or in the freezer (−20°C).
34. Precipitate the DNA by adding 1.4 mL room temperature (∼ 24°C) isopropanol.
35. Close the tubes and mix each sample gently by inverting the tube slowly until completely mixed (precipitated DNA may be observed as shown in Figure 3).

38. Centrifuge the samples for 20 min at 8,000 × *g* 4°C.
39. Remove and discard all ethanol solution with a fine tip pipette.
40. Briefly leave the tube cap open to let the residual ethanol evaporate from the DNA pellet (maximum 30 s).

**CRITICAL:** Do not over-dry a sample. If it happens DNA will not dissolve. This cannot be reverted.

41. Resuspend the pellet in 100 μL 10 mM Tris-HCl Buffer (pH = 8.0). If the pellet does not resuspend, see troubleshooting, problem 3.
42. Store the samples at 4°C at least 12 h to allow for the DNA to be suspended.
43. Transfer samples to clean 1.5 mL Eppendorf Protein LoBind tubes.
44. Store DNA at 4°C until quality control.

**CRITICAL:** Avoid freezing HMW DNA after it has been purified to minimize DNA shearing.

### Quality control

**Timing: 5 h (for 28–56 samples)**

In this step the quality and the quantity of the purified DNA is assessed by its concentration, ratios of absorbance, and degree of fragmentation. The DNA quality control should not be performed before the DNA has had ample time to resuspend (a minimum of 16 h at 4°C).

45. Determine the DNA concentration of the sample by measuring 2 μL sample using the Qubit dsDNA broad range assay.

***Note:*** The ideal concentration is approximately 50 ng/μL and at least 20 μL. For samples with concentrations below 10 ng/μL, see troubleshooting, problem 4.

**Figure 4:**
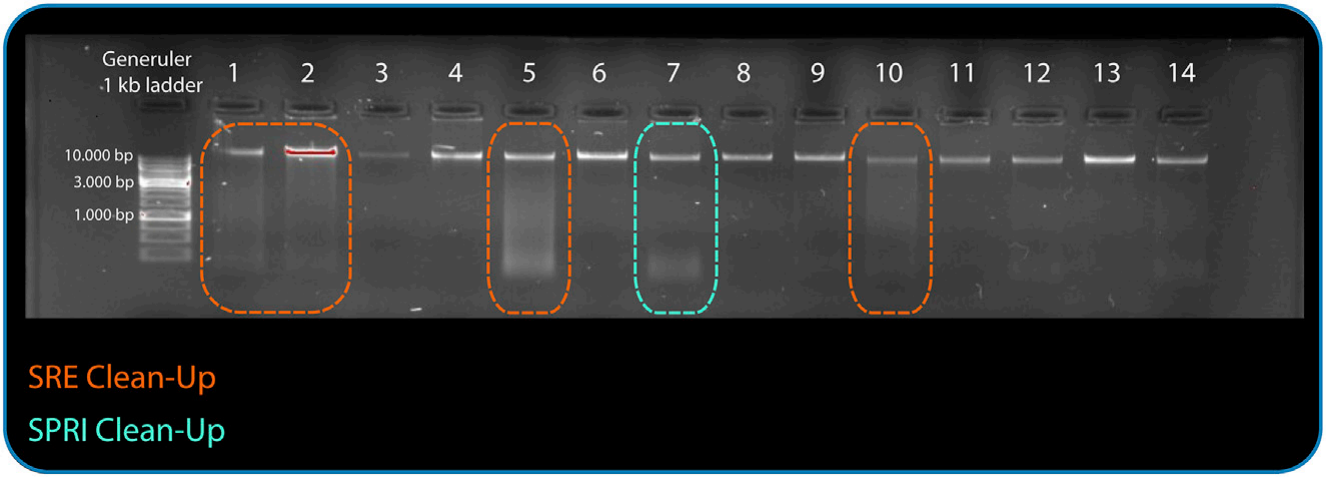
Examples of purified DNA on a 0.8% agarose gel The samples that will need further clean-up based on their degree and type of fragmentation are indicated as either needing 0.4 × SPRI clean-up (in turquoise) or SRE clean-up (in orange). The A260/A280 and A260/230 ratios for the samples are provided in Table 3.
  b. If there are small fragments on the gel (<1.5 kb, lane 7 highlighted with turquoise box in Figure 4) the sample can be cleaned by 0.4 × volume SPRI clean-up.
  c. If the gel shows excessive smearing indicating fragments larger than 3 kb (see lane 1, 2, 5, and 10 highlighted with orange boxes on Figure 4), the sample can be cleaned using a Short Read Eliminator kit (SRE). See troubleshooting, problem 5.
47. Measure the absorbance at 230 nm, 260 nm, and 280 nm and calculate the A260/A280 and A260/A230 absorbance ratios of 2.5 μL of sample using a NanoDrop spectrophotometer.

***Note:*** If the samples fall outside acceptable ranges, that is a 260/280 ratio between 1.8–2.0 and 260/230 ratio between 1.9–2.2 Table 3 see troubleshooting, problem 6.

46. Determine the amount of short DNA fragments by running 100–200 ng of DNA on a 0.8% agarose gel for 20 min at 5 V/cm.
  a. The sample should show a single chromosomal band at the top of the gel as the gel is unable to separate fragments of the length of the average fragment (see example provided in Figure 4).

48. Store samples at 4°C after QC.

### DNA library preparation

**Timing: 2 h (12–16 samples)**

In this step, each sample’s HMW DNA is sheared and has a unique barcode attached using the protocol for the SQK-RBK110.96 kit (Version: RBK_9126_v110_revF 24 Mar 2021) to generate 12–16 DNA libraries that are subsequently pooled into a multiplex sample for sequencing. Ideally, samples with concentrations of ∼ 50 ng/μL (± 10 ng/μL) should be used to promote equimolarity. If the samples do not meet these guidelines, it is advisable to dilute the samples at least one day before preparing the libraries, allowing HMW DNA to be suspended in the final volume at 4°C, and remeasure the DNA concentration as HMW DNA dilutions are highly inaccurate.

***Note:*** The SQK-RBK110.96 protocol has been optimized for the present workflow by modifying the following elements: (1) The initial amount of HMW DNA per sample has been increased from 50 ng to 600 ng and is adjusted to a volume of 27 μL with 10 mM Tris-HCl pH 8.0 (instead of 9 μL and nuclease-free water). (2) To maintain the volume proportion, 3 μL of Rapid Barcode is added to the diluted samples. Hence, the concentration of HMW DNA in relation to barcode has increased by a factor of 4 in comparison with the commercial protocol. (3) For the SPRI clean-up step, the quantity of beads has been modified from 1 × to 0.4 × times the library volume, to remove small fragments and other impurities. In addition, the release of HMW DNA during elution was increased by incubation for 2 min at 50°C.

49. Preheat the thermocycler to 30°C and the heating block to 50°C.
50. Handle kit components as shown in Table 2:

**Table 2:** Conditions for kit reagents (modified from the original Oxford Nanopore SKQ-RBK110.96 protocol)

Reagent	1st: Sample storage/handling	2nd: Sample centrifugation	3rd: Sample mixing
Rapid Barcode plate (RB96)	Keep in the freezer until immediately before use	Spin down briefly	Mix by pipetting
Rapid Adapter F (RAP F)	Keep in the freezer until immediately before use	Spin down briefly	Mix by pipetting
SPRI beads (SPRI)	Place at room temperature (∼ 24°C) for 30 min before use	Spin down briefly	Vortex immediately before use
Sequencing Buffer II (SBII)	Thaw at room temperature (∼ 24°C)	Spin down briefly	Mix by vortexing and spin down briefly
Loading Beads II (LBII)	Thaw at room temperature (∼ 24°C)	Spin down briefly	Mix by pipetting immediately before use
Elution Buffer (EB)	That at room temperature (∼ 24°C)	Spin down briefly	Mix by pipetting
Flush Buffer (FB)	That at room temperature (∼ 24°C)	Spin down briefly	Mix by vortexing and spin down briefly
Flush Tether (FLT)	That at room temperature (∼ 24°C)	Spin down briefly	Mix by pipetting
51. Adjust the volume of each sample following the ratio of 600 ng template DNA to 3 μL of Rapid Barcode:
  a. In PCR tubes, transfer 600 ng genomic DNA per sample adjusting the volume to 27 μL with 10 mM Tris-HCl pH 8.0.
52. Spin down the Rapid Barcode plate in a microplate centrifuge. Add 3 μL of barcode (RB01-96), one for each sample. A multichannel pipette can be used for this step.
  a. Add one barcode per sample.
  b. Seal the PCR tubes with strip lids.
  c. Register samples and corresponding barcodes.
53. Mix the sample tubes gently by flicking and spin down in a microcentrifuge.
54. Incubate in a thermocycler at 30°C for 2 min, and then at 80°C for 2 min.
55. Briefly put the tubes on ice for cooling down.
56. Pool the entire volume from all barcoded samples (libraries) in a 1.5 mL Eppendorf Protein LoBind tube, noting the total volume.
57. Into the tube containing the pooled library, add 0.4 times the volume of resuspended SPRI beads. Mix the pooled sample by flicking the tube.

***Note:*** The ratio can be varied, where 0.4 × removes fragments shorter than 1.5 kb.

58. Incubate tubes on a hula mixer for 5 min at room temperature (∼ 24°C, speed 10 rpm, keep tubes in horizontal position). Meanwhile, prepare 3 mL of fresh 80% (v/v) ethanol in nuclease-free water.
59. Spin down the sample and pellet on a magnet for few minutes.
  a. Keep the tube on the magnetic rack and discard the supernatant.
60. Keep the tube on the magnet and wash the beads with 750 μL of freshly prepared 80% (v/v) ethanol without disturbing the pellet.
  a. Incubate for 30 s.
  b. Remove the ethanol using a pipette and discard.
61. Wash again with 750 μL freshly prepared 80% ethanol for a total of two washes.
62. Briefly spin down and place the tube back on the magnet.
  a. Pipette off any residual ethanol with a fine tip pipette.
  b. Allow to dry for no more than 30 s, do not dry the pellet to the point of cracking.
63. Remove the tube from the magnetic rack and resuspend the pellet in 15 μL Elution Buffer (EB) by gently flicking the tube.
64. Incubate samples for 2 min at 50°C.
65. Incubate for 8 min at room temperature (∼ 24°C).
66. Pellet the beads on a magnet until the eluate is clear and colorless.
67. Transfer 13 μL of eluate into a clean 1.5 mL Eppendorf Protein LoBind tube, while taking care to avoid transferring the beads.
68. Quantify the concentration of the eluted sample, using 2 μL in a Qubit fluorometer.

***Note:*** If concentration is above 100 ng/μL, dilute with EB buffer for a final concentration of 50 ng/μL in a volume of 13 μL. Measure the concentration again using 2 μL of sample in Qubit.

69. Add 1 μL of RAP F to the remaining 11 μL of barcoded DNA.
70. Mix gently by flicking the tube, and spin down.
71. Incubate the reaction for 5 min at room temperature (∼ 24°C).
72. Store the pooled library on ice until ready to load on the flow cell.

### Priming and loading the SpotON flow cell

**Timing: 15 min (for step 73)**

A new flow cell FLO-MIN106D (9.4.1) usually has 1,300–1,500 available nanopores and produces 8–12 Gbases in <24 h with the present method. Although the SQK-RBK110.96 kit has the capacity to sequence 96 samples at a time in a single flow cell, the number of samples is limited by the amount of data necessary per sample. For practical purposes, it is assumed that each sample requires about 100 nanopores to be successfully assembled after sequencing for approximately 16 h. Thus, 12–16 samples can be sequenced per flow cell in <24 h.

73. Allow the flow cell R9.4.1 to come to room temperature (∼ 24°C) for at least 5 min before inserting it into the MinION Mk1B device.
74. Determine the number of active pores in the flow cell by selecting “Check flow cell” in MinKNOW.
75. Open the priming port and check for a small air bubble under the cover.
76. Draw back a small volume to remove any bubble:
  a. Insert a P1000 tip into the priming port.
  b. Turn the pipette’s thumbwheel anti-clockwise until the dial shows a gain of no more than 20–30 μL or until a small volume of storage buffer (yellow color) is entering the pipette tip.

**CRITICAL:** The introduction of air bubbles into the flow cell in any step will permanently damage the integrity of the pore array.

77. Prepare the flow cell priming mix by adding 30 μL of FLT to 1,170 μL of FB, and mix by pipetting, being careful to avoid foaming/air bubbles.

***Note:*** Keep the FB-FLT mix at room temperature (∼ 24°C), not on ice.

78. Load 800 μL of the priming mix into the flow cell via the priming port and wait 5 min.
79. In a new tube, prepare the library for loading as follows (total volume 75 μL):
  a. 37.5 μL SBII sequencing buffer.
  b. 25.5 μL LBII loading beads (mix immediately before use).
  c. DNA library 12 μL.
80. Complete flow cell priming:
  a. Gently lift the SpotON sample port cover to make the SpotON sample port accessible.
  b. Load 200 μL of the priming mix into the flow cell via the priming port (not the SpotON sample port).
81. Mix the prepared library gently by pipetting just before loading to resuspend the loading beads.
82. Load all 75 μL of sample into the flow cell via the SpotON sample port in a dropwise fashion.
83. Turn back the SpotON sample port cover, making sure the bung enters the SpotON port, close the priming port and close the MinION lid.

### Starting the flow cell sequencing run

**Timing: 5 min**

84. Open MinKNOW (v. 21.10.4).
85. Select “Start” then “Start sequencing” and fill in information, as requested:
  a. Selected positions.
    i. Position: select the MinION device.
    ii. Choose an Experiment number and Sample ID.
    iii. Verify the flow cell ID number.
  b. Kit.
    i. Selected kit: SKQ-RBK110.96.
  c. Run options.
    i. Run length: 72 h.
    ii. Bias voltage: -180 mV.
    iii. Adaptive sampling: Off.
  d. Basecalling.
    i. Basecalling: On (High-accuracy basecalling).
    ii. Barcoding: On.
    iii. Trim barcodes: On.
    iv. Detect mid-read barcodes: On.
    v. Alignment: Off.
  e. Output.
    i. FAST5: On (Raw, VBZ, 4,000 reads per file).
    ii. FASTQ: On (GZip, 4,000 reads per file).
    iii. Read filtering: Min Qscore: 7 | Readlength: 1 kb minimum.

***Note:*** After the starting the sequencing run, it is recommendable to monitor the initial result after ca 1 h to ensure that the majority of pores are sequencing samples, and to verify that the fragment length N50 is > 10 kb.

***Note:*** There might be an elevated amount of pores sequencing adapters and short fragments shortly after starting a run: these will generally be depleted, and pores will sequence an increasingly high fraction of sample DNA until a maximum is reached after several hours.

86. Let the data generation continue ∼ 16 h and the next day check the amount of data generated per barcode.
  a. In total 8–12 Gbases should be generated, or 500–800 Mbases per sample.

***Note:*** It is very hard to achieve even barcode distribution, so generating excess data will ensure that libraries with less data will assemble. Between 10% and 20% of data will not have a barcode assigned.

87. Continue to initial assembly of data, freeing up the flow cell to sequence new samples.

### Initial de novo assembly of data

**Timing: 4 h (for step 88)**

For complete *de novo* assembly of an Actinobacteria genome using Nanopore sequencing, a coverage > 50 ×, and read N50 > 10 kb is desirable, though some genomes can assemble from less data or shorter reads.

88. Open a terminal and navigate to the run folder. Then in each barcode folder, run:

>flye --nano-raw ∗fastq.gz -i 0 -t 12 -o flye

89. Check stats inside the generated folder, do:

>grep -P ‘Total read length|Reads N50’ flye.log

90. Inspect the coverage, circularity, and edges on each generated contig. A complete contig is either circular or is denoted with a ‘∗’ before and after the repeat-graph path of each contig (see Figure 6):

>cat assembly_info.txt

91. Visualize the assembly repeat-graph using Bandage:

>Bandage load assembly_graph_gfa

***Note:*** If the assembly is incomplete, see troubleshooting, problem 7.

### Illumina data generation and hybrid assembly

**Timing: 4 h for hybrid genome assembly (for step 94)**

To achieve a high quality of assembly, it is necessary to supplement the Oxford Nanopore 9.4.1 data with Illumina data, and then use this data to polish the complete genome sequence generated with the long-read data.

92. Generate or order Illumina library and sequencing at a service provider.
  ***Note:*** As Illumina input DNA requirements are much more flexible for Illumina data than for nanopore data, commercial service providers can easily produce the needed amount and quality of data.
  a. Use a protocol without transposase shearing, and with a low number of PCR cycles, such as the NEB Next Ultra DNA Library Prep Kit with only 6 PCR cycles.
  b. Sequence with 2 × 150 nt paired-end chemistry and coverage of ∼ 50 for each genome on a NovaSeq Illumina machine.
    ***Note:*** These suggestions are based on the high GC content of Actinobacteria, which if not followed will lead to genomic areas not covered by the Illumina data and thus left unpolished.

93. Generate the hybrid assembly:
  a. Use the Flye assembler^3^ with a total of five rounds of polishing with the long-read data.
  b. Polish the assembly, first with Polypolish^5^ and subsequently POLCA,^6^ as suggested in.^5^

***Note:*** Using Polypolish is particularly important for genomes like *Streptomyces*, where a large, inverted repeat carrying biosynthetic gene clusters will otherwise have approximately 1 insertion or deletion error per 1,000 nt sequence, which severely limits analysis.

***Note:*** Generation of Illumina data in collaboration with a service provider is generally expected to take several weeks.

94. Open a terminal and navigate to the run folder. For each sample run:

>flye --nano-raw ∗fastq.gz -i 5 -t 12 -o flye

95. For the first round of short read polishing of each genome using Polypolish, create a folder ‘illumina’ inside each strain folder, copy the illumina data there, and run:

>bwa index flye/assembly.fasta

>bwa mem -t 12 -a flye/for_polishing.fa illumina/R1.fq.gz > alignments_1.sam

>bwa mem -t 12 -a flye/for_polishing.fa illumina/R2.fq.gz > alignments_2.sam

>polypolish_insert_filter.py --in1 alignments_1.sam --in2 alignments_2.sam --out1 filtered_1.sam --out2 filtered_2.sam

>polypolish flye/assembly.fasta filtered_1.sam filtered_2.sam > polypolished.fasta

96. For the second round of short read polishing of each genome using POLCA, run:

>polca.sh -a polypolished.fasta -r 'illumina/R1.fq.gz illumina/R2.fq.gz' -t 12

97. The file ‘polypolished.fasta.PolcaCorrected.fa’ will contain the polished genome sequence.
98. Verify the quality of the assembly using BUSCO (v. 5.1.2).

>busco -i polypolished.fasta.PolcaCorrected.fa --mode geno --lineage_dataset actinobacteria_class_odb10 -o busco -c 12

***Note:*** the fragmented and incomplete BUSCO genes should be <5% combined, and the number of duplicated BUSCO genes should be < 20. Some duplicated BUSCO genes are expected and likely a result of self-resistance.^11^

## Expected outcomes

### HMW DNA extraction

The main goal after the lysis and purification protocol is to obtain HMW DNA of sufficient concentration and quality for the sequencing of the complete bacterial genome. Following precipitation of HMW DNA in most cases the molecules can be visually confirmed (see Figure 3), providing an indication of the success of the extraction and high concentration, although not seeing DNA is not an indication of failed extraction.

Due to the heterogeneity between samples, the concentrations will also differ. Generally, sample concentrations range from 20–300 ng/μL. Table 3 shows the quality of 14 samples processed according to the protocol.

**Table 3:** Absorbance ratios of 14 samples

Name	Qubit DNA concentration	260/280	260/230
Sample 1	497.2 ng/μL	1.86	2.02
Sample 2	225.3 ng/μL	1.83	1.97
Sample 3	3.6 ng/μL∗	2.1∗	1.46∗
Sample 4	191.5 ng/μL	1.88	2.11
Sample 5	603.1 ng/μL	1.88	2.05
Sample 6	195.4 ng/μL	1.87	2.04
Sample 7	59.1 ng/μL	1.88	1.92
Sample 8	131.2 ng/μL	1.86	2.01
Sample 9	16.6 ng/μL	1.99	1.73∗
Sample 10	335.8 ng/μL	1.86	2.04
Sample 11	10.7 ng/μL	2.08∗	1.51∗
Sample 12	21.6 ng/μL	1.69∗	1.11∗
Sample 13	281.9 ng/μL	1.87	1.97
Sample 14	37.7 ng/μL	1.93	1.86∗

Ratios outside of the desired range are indicated with an asterisk (∗). The fragmentation of the same samples is depicted in Figure 4

In terms of purity, a sample should have an A260/A280 ratio close to ∼ 1.8 and an A260/A230 ratio above 2.0. We accept 260/280 ratios between 1.8–2.0 and 260/230 ratios between 1.9–2.2. If the sample falls outside this range, see troubleshooting, problem 6. An example is provided in Table 3.

**Figure 5:**
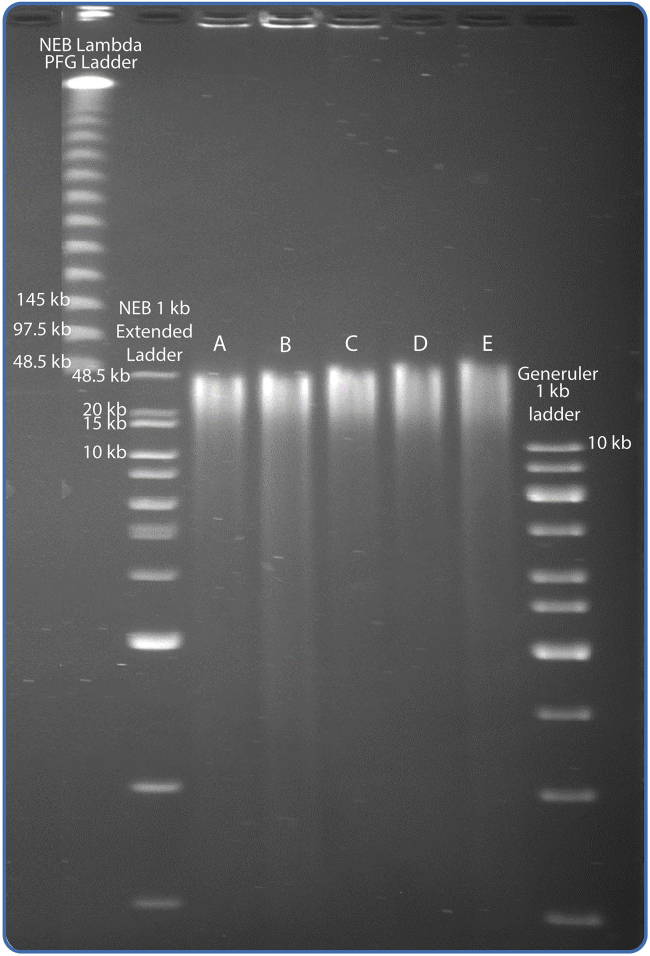
PFGE 0.8% agarose gel Each lane from A-E is loaded with a sample of HMW DNA that have passed the quality control and have an approximate concentration of 50 ng/μL. It is not necessary to check samples using PFGE, this figure is an illustration of the fragment lengths expected with this protocol.

Samples that pass quality control should have HMW DNA fragments of approximately 50 kb in length (as reflected in the pulsed-field gel electrophoresis picture in Figure 5).

### Assembly result

**Figure 6:**
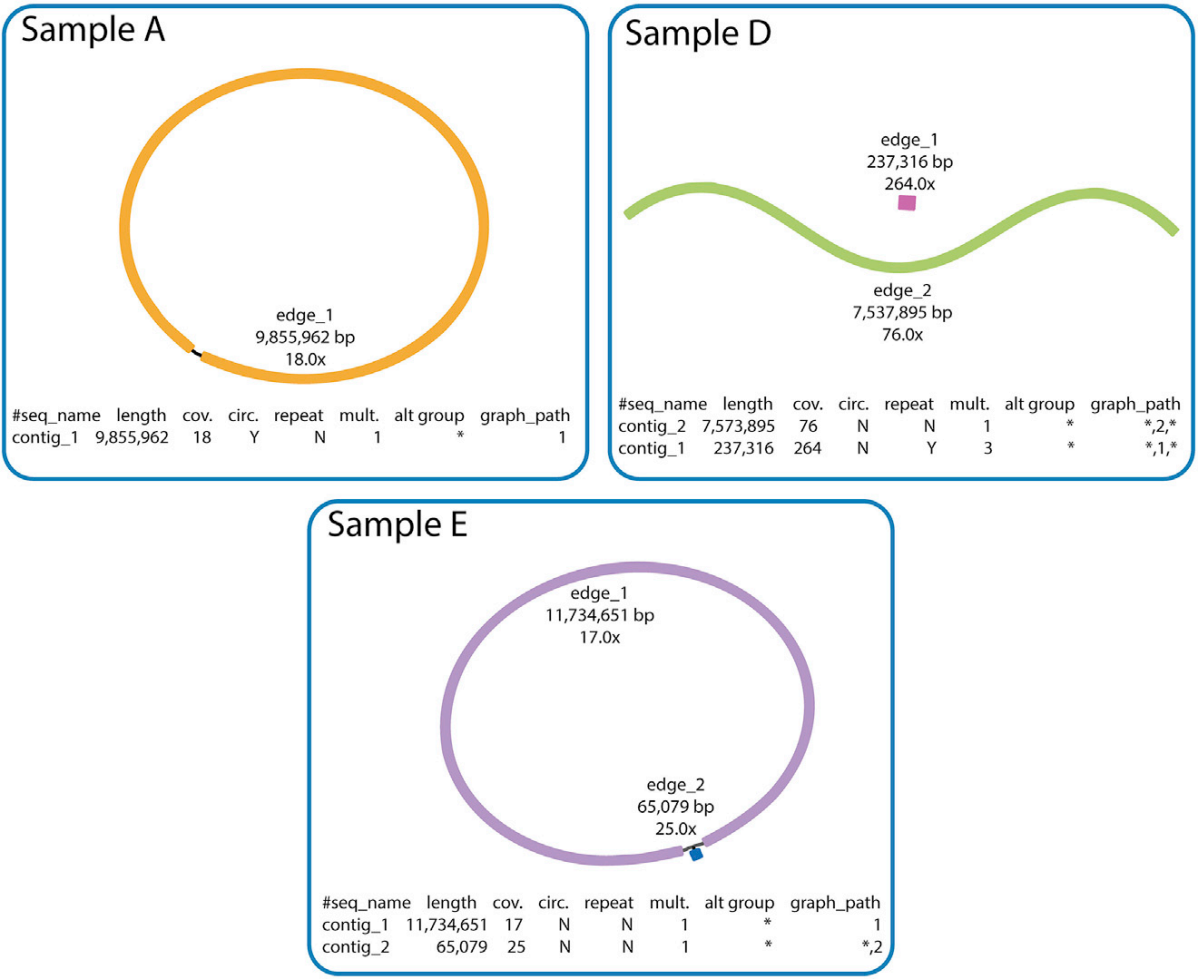
Expected assembly outcomes in Actinobacteria visualized using Bandage In sample A, the chromosome structure is circular. Sample D consists of a linear chromosome and a linear plasmid. Sample E is composed of a linear chromosome with inverted repeats at the end. Further description of the samples is provided in Table 4.

Obtaining a complete assembled genome of high quality is the expected last result. This should be a single contig for the bacterial chromosome, additionally, there may be other contigs for plasmids, one contig per replicon. As previously mentioned, *Streptomyces* usually have linear genomes with inverted repeat and telomeric structures (Figure 6).

Generally, it should be possible to assemble complete genomes from samples with a coverage of 50 × (this can be found at the end of the log file produced by Flye and in the repeat-graph file produced by Flye) and with an N50 of > 10 kb (Table 4, Figure 6).

**Table 4:** DNA concentration and read statistics of samples A-E

Name	Qubit DNA concentration (ng/μL)	Total bases	N50
Sample A	44.6	234,289,864	15,071
Sample B	56.1	435,657,431	14,935
Sample C	52.2	190,157,556	14,058
Sample D	53.3	756,392,161	18,653
Sample E	86.5	226,159,037	22,153

There are two genomic topologies in Actinobacteria, linear and circular. In the assembly repeat graph, these topologies are expected to be represented in one of the following ways:

Circular chromosome: Flye generates a repeat-graph path of a single contig from the chromosome with a single edge, marked as circular (see Sample A, Figure 6).

Linear chromosomes without inverted repeats at the ends: Flye describes the repeat-graph path in a single contig of a single edge with a (∗) symbol that denotes a terminal graph node. An example of this type can be seen in Sample D, Figure 6, which also has a linear plasmid placed in a second contig, note that it is multiplied three times, indicating a copy number of 3.

***Note:*** The telomeric chromosome ends can be present even if the repeat graph does not show them if they are resolved during the assembly, for example if the read length greatly exceeds the IR and telomeric repeats.

Linear chromosomes with inverted repeat and telomeric structures at the end: Flye, often, places the inverted repeat in the repeat-graph on a separate contig instead of in both ends of the chromosome (see Sample E, Figure 6, troubleshooting, problem 7).

***Note:****Streptomyces* usually have linear chromosomes with inverted repeat and telomeric structures at the end of the chromosome.

## Limitations

This protocol should work for all cultured Actinobacteria. However, occasionally, it is not possible to obtain a complete genome sequence (one replicon = 1 contig) for a strain, for example, if repeats much greater than the read length are present. The entire genome sequence will be there, just fragmented, which can limit subsequent analysis.

A limiting factor for the throughput of this protocol is the number of mortars and pestles available: a clean mortar and pestle is needed per sample.

For *Streptomyces* strains, the genome is often linear with inverted repeat chromosome ends. This structure will often not be resolved in the assembly repeat graph and is often not fully assembled or wrongly assembled in the final Flye output. An assembly with repeats longer than the read length will generally be incomplete.

This protocol does not adapt to the Short Real Eliminator protocol, and in such, does not cover troubleshooting connected to it.

It is important to note, that while long-read Oxford Nanopore reads will enable a complete genome assembly, it currently does have a higher error-rate than short-read sequencing. Therefore, supplementing with short reads for polishing the assembly will provide both a complete and highly accurate assembly.

## Troubleshooting

### Problem 1

Not enough material in the culture.

Insufficient biomass (<150–250 mg) in the initial pellet can lead to little or no DNA being extracted in the end.

### Potential solution

• Increase the culture volume used to pellet biomass.
• Increase the incubation time of the strain before harvesting.
• Inoculate several copies of the strain in question and pool them before harvesting.

If the strain exhibits little to no growth at all, change the media/conditions used to grow the strain. Conditions, which have worked previously, for instance when the strain was isolated (changed temperature, reduced rpm or different media) are ideal options for this. Alternatively, consult Practical Streptomyces Genetics^9^ for guidance.

### Problem 2

Clogged column.

During the extraction process, if after loading the sample or any other reagent the column becomes clogged, and the sample is not passing through.

### Potential solution

• Check the viscosity of the sample. If it is high, recover the volume loaded on the column and dilute the sample with B3 buffer.
• The clogging can be overcome by adding slight positive and constant pressure with air from a syringe (10 mL). It is important to exert as little pressure as necessary to avoid shearing of DNA as much as possible. Stop exerting pressure and remove the syringe from the column before the entire volume has passed through the column. Introducing air to the filter must be avoided. If there was a need to applying pressure into the column, this might be needed to be repeated in the following steps as well.

**Caution:** Putting additional pressure on the column puts physical stress on the DNA, which can result in further fragmentation of the DNA.

### Problem 3

DNA Pellet does not dissolve.

If the DNA pellet was excessively dried, it will not easily dissolve in the TA Buffer.

### Potential solution

• If time is ample; incubate the sample at 4°C for several days or weeks. It takes time for HMW DNA to dissolve and homogenize, especially after over-drying.
• If time is important: Incubate the sample at 50°C and gently flick it occasionally.
• If the DNA pellet was overdried in any step, it may be impossible to sufficiently resuspend. In this case, repeat the entire extraction.

### Problem 4

Low DNA concentration.

A low final concentration (<10 ng/μL) indicates that an insufficient amount of biomass was used at the start of the extraction or that DNA was lost during the purification step (usually during the precipitation).

### Potential solution

• Repeat the extraction of the sample. If the sample was not ground in LN the first time, do that now.
• Sequence it anyway: If the sample otherwise passed quality control it can be sequenced, but ideally together with other samples of similar concentration, as this will otherwise skew barcode distribution. A decrease in throughput should be expected and can be compensated for by adjusting the pooling of samples.
• Perform a 1 × volume SPRI clean-up and elute in a reduced volume.

### Problem 5

Smears on gel.

A smear on the agarose gel indicates fragmentation of the sample or overloading of the agarose gel.

### Potential solution

• If the DNA concentration is higher than 200 ng/μL, dilute the sample in question ∼ 50 ng/μL and incubate for at least 12 h at 4°C. Repeat the agarose gel electrophoresis.
• Perform an SRE clean-up to remove fragments below 10 kb. Note that 25%–75% of DNA is lost during the SRE protocol.

### Problem 6

Poor A260/A280 or A260/230 ratios.

A low A260/A280 can indicate contamination by proteins, while a low A260/A230 can indicate salt contamination.

### Potential solution

• Repeating the extraction process, taking care of the sample in question. If the sample was not ground in LN the first time, do that now.
• Exchanging the buffer by performing a 1.0 × SPRI clean-up.

### Problem 7

Incomplete genome assembly.

Occasionally, a genome will not fully assemble even if the read length and coverage are good (N50 > 10 kb, cov >50 ×).

### Potential solution

• Use Filtlong (https://github.com/rrwick/Filtlong) to remove the shortest and lowest quality reads. We suggest removing 30%, 50%, 75% or even 90% of data, aiming to remove the largest amount of data while still having a coverage of 50. When all replicons are assembled into contigs where any assembly repeat graph branching can be explained by biology (i.e., the inverted repeats of *Streptomyces*, see Figure 5, sample E), it is indicative of a completely assembled genome, as long as the coverage is still substantial. In some cases, a coverage <50 can yield a complete genome.
• Use a different genome assembly program. Even though Flye performs consistently well, using another assembly program might help alleviate an assembly issue. We suggest using one of the assemblers benchmarked in Wick and Holt.^12^
• Generate more data. After stopping a sequencing run, the flow cell can be washed and reloaded with sequencing libraries from samples, which did not fully assemble. This will yield a higher coverage, which might be necessary to completely assemble a genome.
• For *Streptomyces* strains, which generally have linear genomes, often with long terminal inverted repeats, the chromosome is often split into two contigs in the final Flye assembly, or the inverted repeat is incorrectly placed on its own contig in addition to in both ends of the chromosome. If this problem is not resolved by using a different assembly program, we suggest to manually concatenate the repeat graph edges and verify their orientation by mapping the Nanopore reads and inspecting the edge borders closely. Then, all three polishing steps (Flye, Polypolish, POLCA) needs to be repeated on the concatenated genome.

## Resource availability

### Lead contact

Further information and requests for resources and reagents should be directed to and will be fulfilled by the lead contact, Tue Sparholt Jørgensen (tuesparholt@gmail.com).

### Materials availability

This study did not generate new unique reagents.

## Data Availability

Any additional information required to reanalyze the data reported in this paper is available from the lead contact upon request. This study did not generate code beyond the described assembly and quality control command-line interface commands.
